# METTL15 introduces N4-methylcytidine into human mitochondrial 12S rRNA and is required for mitoribosome biogenesis

**DOI:** 10.1093/nar/gkz735

**Published:** 2019-09-06

**Authors:** Lindsey Van Haute, Alan G Hendrick, Aaron R D’Souza, Christopher A Powell, Pedro Rebelo-Guiomar, Michael E Harbour, Shujing Ding, Ian M Fearnley, Byron Andrews, Michal Minczuk

**Affiliations:** 1 Medical Research Council Mitochondrial Biology Unit, University of Cambridge, Hills Road, Cambridge CB2 0XY, UK; 2 STORM Therapeutics Limited, Moneta Building, Babraham Research Campus, Cambridge CB22 3AT, UK; 3 Graduate Program in Areas of Basic and Applied Biology (GABBA), University of Porto, Rua Alfredo Allen 208, Porto 4200-135, Portugal

## Abstract

Post-transcriptional RNA modifications, the epitranscriptome, play important roles in modulating the functions of RNA species. Modifications of rRNA are key for ribosome production and function. Identification and characterization of enzymes involved in epitranscriptome shaping is instrumental for the elucidation of the functional roles of specific RNA modifications. Ten modified sites have been thus far identified in the mammalian mitochondrial rRNA. Enzymes responsible for two of these modifications have not been characterized. Here, we identify METTL15, show that it is the main N4-methylcytidine (m^4^C) methyltransferase in human cells and demonstrate that it is responsible for the methylation of position C839 in mitochondrial 12S rRNA. We show that the lack of METTL15 results in a reduction of the mitochondrial *de novo* protein synthesis and decreased steady-state levels of protein components of the oxidative phosphorylation system. Without functional METTL15, the assembly of the mitochondrial ribosome is decreased, with the late assembly components being unable to be incorporated efficiently into the small subunit. We speculate that m^4^C839 is involved in the stabilization of 12S rRNA folding, therefore facilitating the assembly of the mitochondrial small ribosomal subunits. Taken together our data show that METTL15 is a novel protein necessary for efficient translation in human mitochondria.

## INTRODUCTION

Mammalian mitochondrial DNA (mtDNA) encodes 13 polypeptides that are essential subunits of the oxidative phosphorylation (OxPhos) system. MtDNA also contains the genes for all 22 mitochondrial (mt-) tRNAs and two mt-rRNAs (small 12S and large 16S). The maintenance and expression of the mitochondrial genome is entirely dependent on proteins encoded in the nucleus, which upon translation on cytoplasmic ribosomes are imported into mitochondria. Expression of mitochondrial genes shows very distinct differences as compared to their nuclear-encoded counterparts, particularly with respect to protein synthesis. Mammalian mitoribosomes differ considerably from other ribosomes as far as architecture and composition is concerned, with the key differences being the reversed protein:RNA mass ratio and many novel, mitochondria-specific protein components. Mammalian mitoribosomes are composed of the mtDNA-encoded 12S and 16S rRNA and 82 nucleus-encoded mitoribosomal proteins (MRPs) (1–3). The 5S rRNA conventionally present as a third rRNA component in bacterial and cytoplasmic ribosomes has been replaced by mt-tRNA^Val^ (e.g. human, rat) or mt-tRNA^Phe^ (e.g. pig, cow) in the central protuberance of the mitochondrial large ribosomal subunit (mt-LSU) (1,4–6). Although the structure of the mammalian mitoribosome has been determined, little is known about the assembly mechanism. Given the divergence of the mammalian mitoribosome from its ancestor, its assembly pathway is likely to be considerably different from its bacterial counterpart, implying the presence of mitochondria-specific regulatory factors. Recently, a series of pulse-labelled SILAC experiments has given the first important insight into the kinetic appearance of newly synthesized MRPs in the assembled mitoribosome (7), although the exact mechanism is still to be unravelled.

In all kingdoms of life, rRNAs undergo multiple post-transcriptional modifications, which are crucial for ribosome biogenesis and/or efficient translation. In comparison with bacterial and eukaryotic cytoplasmic rRNAs, the number of modifications observed in mitochondrial rRNAs is low. Only ten modified sites have been identified in 12S and 16S mt-rRNA thus far, whereas >200 and >30 modified nucleotides have been detected in eukaryotic cytoplasmic and bacterial ribosomes, respectively. Mammalian large 16S rRNA has three 2′-*O*-ribose methylations, Gm1145, Um1369 and Gm1370 (m. 2815G, m.3039T, m.3040G, respectively, human mtDNA numbering), catalysed by MRM1, MRM2 and MRM3, respectively (8–10) and a base methylation at position m^1^A947 (m.2617A), catalysed by TRMT61B (11). Furthermore, mammalian 16S rRNA contains one pseudouridine (ψ) at position 1397 (m.3067T) (12), which is predicted to be introduced by RPUSD4 (13,14). Five base methylations have been identified in the small 12S rRNA (15). Positions m^6^_2_A936 and m^6^_2_A937 (m.1592A and m.1593A) of the mammalian 12S rRNA are methylated by TFB1M (16,17), while the C5 methylation of C841, m^5^C841 (m.1488C), is catalysed by NSUN4 (18). The enzymes responsible for the remaining two methylations detected in mammalian 12S rRNA, m^5^U429 (m.1076T) and m^4^C839 (m.1486C) are still awaiting identification.

Methylation of the small subunit (SSU) rRNA position equivalent to the 12S mt-rRNA m^4^C839 is strongly conserved in bacteria (m^4^C1402 in the SSU 16S rRNA in *Escherichia coli*) (19–22). The enzyme responsible for m^4^C1402 in *E. coli* has been identified as RsmH (also known as mraW) and is an *S*-adenosyl methionine (SAM)-dependent methyltransferase. RsmH shows sequence homology with mammalian methyltransferase-like family (METTL) members. Proteins of this family are involved in multiple cellular functions. For example, the METTL3/METTL14 complex and METTL16 methylate N6-adenosine on mammalian nuclear-encoded RNA (23), METTL1 methylates N7-guanosine of yeast and human tRNA (24–26), and METTL12 is a mitochondrial methyltransferase that modifies citrate synthase (27).

We have recently shown that defects in the nucleotide modification of mt-rRNA can lead to a human disorder of mitochondrial respiration (28). Here we demonstrate that a previously uncharacterized protein, encoded in the nucleus by *METTL15*, is localized in mitochondria in human cells and is required for the introduction of N4-methylcytidine at position 839 of human 12S mt-rRNA. Our study also shows that METTL15 is necessary for efficient mitochondrial protein synthesis and mitoribosome biogenesis. These findings on the role of METTL15 provide new insights into the mitoribosome assembly process and, in the long-term, can help pave the way for the development of future mechanism-based therapies for mitochondrial diseases.

## MATERIALS AND METHODS

### Maintenance and transfection of mammalian cell lines

HeLa cells, used for immunofluorescence and siRNA mediated knockdown of METTL15 were grown at 37°C in a humidified atmosphere with 5% CO_2_ in high-glucose DMEM supplemented with 10% foetal bovine serum.

The Flp-In T-Rex HEK293T cell line was used to generate a doxycycline-inducible expression of METTL15.FLAG.STREP2. The full-length METTL15 cDNA construct (IMAGE clone: 4657323 with sequence corrected using site-directed mutagenesis) was cloned into a pcDNA5/FRT/TO plasmid encoding FLAG.STREP2-tag as previously described (29). The oligonucleotides used for cloning can be found in Supplementary Table S1. Cells were grown in the same medium described above, supplemented with selective antibiotics hygromycin (100 μg/ml) and blasticidin (15 μg/ml). Wild type and METTL15 knockout HAP1 cells were grown in Iscove's Modified Dulbecco's Medium (IMDM) supplemented with 10% fetal bovine serum or IMDM medium for SILAC supplemented with Arg, Lys and Pro and 10% dialysed FCS (Thermo Scientific HyClone). METTL15 was knocked-out in HAP1 cells using CRISPR/Cas9 by Horizon Discovery.

### Cell fractionation

Fractionation experiments were performed by differential centrifugation as previously described (29), omitting the sucrose gradient step. Briefly, following trypsinisation, cells were washed with PBS and resuspended in hypotonic buffer containing 20 mM HEPES (pH 7.8), 5 mM KCl, 1.5 mM MgCl_2_ with 1 mg/ml BSA and protease inhibitors (Roche), incubated on ice for 10 min and homogenized with a Balch homogeniser with a 10 μm clearance ball bearing. Next, two-thirds of total homogenate volume of 2.5× MSH buffer (525 mM mannitol, 175 mM sucrose, 20 mM HEPES (pH 7.8), 5 mM EDTA and protease inhibitor cocktail) was immediately added. 1× MSH buffer was added up to 30 ml. Next differential centrifugation was used to obtain the different cell fractions (1000 × g for 10 min to remove cell debris and nuclei, next for 10 min at 10 000 × g to pellet the mitochondria).

### Immunodetection of proteins

The immunofluorescence analysis was performed essentially as described previously (30). Briefly, HeLa cells were grown on coverslips and transiently transfected with METTL15.FLAG.STREP2 using Lipofectamine 2000 (Thermofisher) for 24 h and fixed with 4% formaldehyde solution. After permeabilization with 1% Triton X-100, cells were incubated with the following antibodies: rabbit anti-FLAG (Sigma-Aldrich, F3165, 1:200), mouse anti-TOM20 (Santa Cruz Biotechnology, sc-11415, 1:500) followed by Alexafluor-488 anti-mouse or Alexafluor-594 anti-rabbit (Molecular Probes). Mounting medium used was ProLong Gold Antifade Mountant with DAPI (Molecular Probes).

For western blot analysis, 20–30 μg of extracted proteins were loaded on SDS-PAGE 4–12% bis–tris gels (Life Technologies) and transferred onto a membrane using iBlot 2 Dry Blotting System (Thermo Fisher Scientific). The following antibodies were used: rabbit anti-METTL15 (Sigma-Aldrich, HPA048994, 1:1000), mouse anti-TOM22 (Abcam, ab10436, 1:2000), mouse anti-GAPDH (Abcam, ab9484, 1:5000) mouse anti-FLAG (Sigma, F3165, 1:1000), Total OXPHOS Human WB antibody cocktail (Abcam, ab110411, 1:1000), mouse anti-Beta actin (Sigma-Aldrich, A1978, 1:75 000), mouse anti-mtSSB1 (a kind gift of Prof. D. Kang, 1/4000), rabbit anti-uL3m (Sigma-Aldrich, HPA043665, 1:2000), rabbit anti-bL112m (Proteintech, 14795-1-AP, 1:2000), rabbit anti-uS17m (Proteintech, 18881-1-AP, 1:1500), rabbit anti-mS40 (Proteintech, 16139-1-AP, 1:1500), mouse anti-mS29 (Abcam, ab11928, 1:1000), rabbit anti-uL223m (Proteintech, 11706-1-AP, 1:1000), mouse anti-SDHB (Life Technologies, 459230, 1:500).

### Gene silencing by RNAi

Stealth siRNAs were obtained from Thermo Fisher Scientific and transfected into HeLa cells using Lipofectamine RNAiMAX Reagent (Thermo Fisher Scientific). Sequences can be found in Supplementary Table S1.

### Mitochondrial DNA copy number analysis

Total cellular DNA was isolated from HAP1 cells using a DNeasy Blood and Tissue kit (Qiagen) according to the manufacturer's recommendations. Copy numbers of mtDNA were measured by quantitative PCR as described previously (31). Primer and probe sequences can be found in Supplementary Table S1.

### RNA extraction and RT-qPCR

Measurements of RNA steady-state levels by RT-qPCR was performed as described previously (32). Briefly, RNA was extracted with TRIzol reagent (Thermo Fisher Scientific) and treated with TURBO DNase (Ambion) according to manufacturer's instructions. 1 μg RNA was reverse transcribed using Omniscript RT kit (Qiagen) with 0.5 μM random hexamers and 0.5 μM oligo dT primers. RT-qPCR primer and probe sequences can be found in Supplementary Table S1.

### ^35^S-methionine metabolic labelling of mitochondrial proteins

To label newly synthesised mtDNA-encoded proteins the previously published protocol was used (33). Briefly, exponentially growing cells were incubated in methionine/cysteine-free medium for 10 min before the medium was replaced with methionine/cysteine-free medium containing 10% dialysed FCS and emetine dihydrochloride (100 μg/ml) to inhibit cytosolic translation. Following a 20 min incubation, 120 μCi/ml of [^35^S]-methionine (Perkin Elmer) was added and the cells were incubated for 30 min. For the pulse-chase experiments, the labelling medium was removed and cells were allowed to grow for 7 and 14 h in normal growth medium before collection. After washing with PBS, cells were lysed and 30 μg of protein was loaded on 10–20% Tris-glycine SDS-PAGE gels. Products were visualized and quantified with a PhosphorImager system with ImageQuant software.

### RNA mass spectrometry

Mitochondrially-enriched RNA was digested to component nucleosides with an enzyme cocktail of benzonase, phosphodiesterase and alkaline phosphatase (Merck), all used as according to the manufacturer's instructions. Nucleosides were separated by reverse phase liquid chromatography - eluent A was 0.1% (v/v) formic acid in water, and eluent B was 0.1% (v/v) formic acid in acetonitrile, and a non-linear gradient of 2–15% B resolved nucleosides across a Waters Acquity HSS T3 C18 column. The eluent was sprayed into a Sciex 4500 triple quadrupole mass spectrometer and characterized by tandem mass spectrometry using a multiple reaction monitoring approach. Injection amounts were normalized by internal calibration with isotopically-labelled uridine and quantification performed using external calibration of a range of nucleoside standards.

### Targeted RNA bisulfite sequencing

Bisulfite conversion of 2 μg DNase treated RNA was performed using the Imprint DNA Modification kit (Sigma). The reaction mixture was incubated for three cycles of 90°C for 5 min and 60°C for 1 h. Following desalting with Micro Bio-spin 6 chromatography columns (Bio-Rad) twice, RNA was desulfonated by adding an equal volume of 1 M Tris (pH 9.0) and incubated for 1 h at 37°C. After ethanol precipitation, reverse transcription was performed with specific primers (Superscript II, Life technologies). After the first stage PCR with overhang primers (Supplementary Table S1), excess primers were removed with Ampure XP beads. An 8 cycles second round PCR was performed with indexed primers (Nextera XT), followed by another clean-up with Ampure XP beads. Quality and concentration were assessed with a D1000 Screentape for TapeStation (Agilent Genomics). Libraries were subjected to high-throughput sequencing using the Illumina MiSeq platform. After quality trimming and 3′ end adaptor clipping with Trim_Galore!, reads longer than 20 nt were aligned to a computationally bisulfite-converted human reference genome (GRCh38) with Bismark (34).

### Complementation of METTL15 KO lines with WT or mutant METTL15 cDNA

The METTL15 WT cDNA described above was subjected to site-directed mutagenesis to encode predicted catalytically inactive protein. Primer sequences can be found in Supplementary Table S1. PCR products were cloned into the lentiviral vector pWPXLd-ires-PuroR (derived from pWPXLd, Addgene #12258). Human 293T cells were plated at 2.5–3 × 10^6^ cells per 10 cm plate, 24 h before cotransfection with 10 μg of METTL15 cDNA-containing transfer vector, 6.55 ug of second-generation packaging plasmid (psPAX2) and 3.5 μg of envelope plasmid (pMD2.G) using FuGENE 6 Transfection Reagent (Roche). Viral particles were collected 24 h after transfection. METTL15 KO HAP1 cells were transduced with lentiviral particles carrying the WT METTL15 gene or a putative catalytically inactive mutant. Cells were selected with puromycin (0.5 μg/ml) for 14 days and bulk populations of cells were subjected to further analysis.

### Protein co-immunopurification

Mitochondria were isolated by differential centrifugation from HEK293T cells expressing a tagged METTL15 protein. Pelleted mitochondria were resuspended in lysis buffer and immune affinity purification was performed with an M2 affinity gel kit with elution of the native protein conducted with peptide following the manufacturer's protocol (Sigma-Aldrich).

### Analysis of the mitochondrial ribosome profile on sucrose density gradients

Wild type or METTL15 KO HAP1 cells were grown in ‘heavy’, containing ^15^N and ^13^C-labelled Arg and Lys, or ‘light’ labelled media. Both cell lines were pooled together and mitochondrial isolation was performed as described above, before lysis and loading on a linear sucrose gradient (10–30%) in 50 mM Tris–HCl (pH 7.2), 10 mM Mg(OAc)_2_, 40 mM NH_4_Cl, 25 mM KCl and centrifuged for 2 h 15 min at 100 000 × g max at 4°C. Fractions (of 100 μl each) were collected, and 30 μL portions were precipitated with 20× volume of ethanol overnight and precipitants digested with 1:100 (w/w) of trypsin in 50 mM NH_4_HCO_3_ overnight. Liquid chromatography-tandem mass spectrometry (LC–MS/MS) analysis of the peptides was carried out using a Thermo Scientific Orbitrap LTQ XL mass spectrometer and a Proxeon EasyLC nanoscale chromatograph. Spectral data was analysed by Thermo Proteome Discoverer software which directed firstly protein identification with Matrix Science Mascot software (configured to query the human Uniprot database) and then performed relative quantification of peptide precursors. Custom Python and R scripts were written and used to separate results for heavy and light-isotope-labelled peptides into control and treatment batches, and from them to identify peptides to represent relative protein abundance and create relative abundance profiles across the sucrose gradient.

### Structural modelling of human METTL15

Amino acid sequence for METTL15 was retrieved from Uniprot (A6NJ78) (Supplementary Figure S2: Transcript ID ENST00000407364.7) and modelled using *E. coli* high resolution crystal structures for methyltransferase Rsmh (PDB:3TKA) as template using the Swiss-Model homology modelling server. AdoMet from the bacterial structure and the residues involved in the AdoMet-binding in the *E. coli* methyltransferase were identified and mapped onto the aligned structures to analyse conservation of catalytically important residues. Structure visualization and mapping of residues was performed using PyMOL (35).

### Evaluation of structural proximity

The structure of the human mitoribosome (PDB 3J9M (1,4)) was inspected for the closest ribosomal proteins to 12S C839. First, the central position of residue C839 was determined using all of its atoms (20 atoms), and the resulting coordinate was used to calculate the distance to all atoms from the protein components of the mitoribosomal small subunit. Individual distances between atoms were determined by inspection of the structure using PyMOL (35).

## RESULTS

### METTL15 is localized in human mitochondria

**Figure 1.**
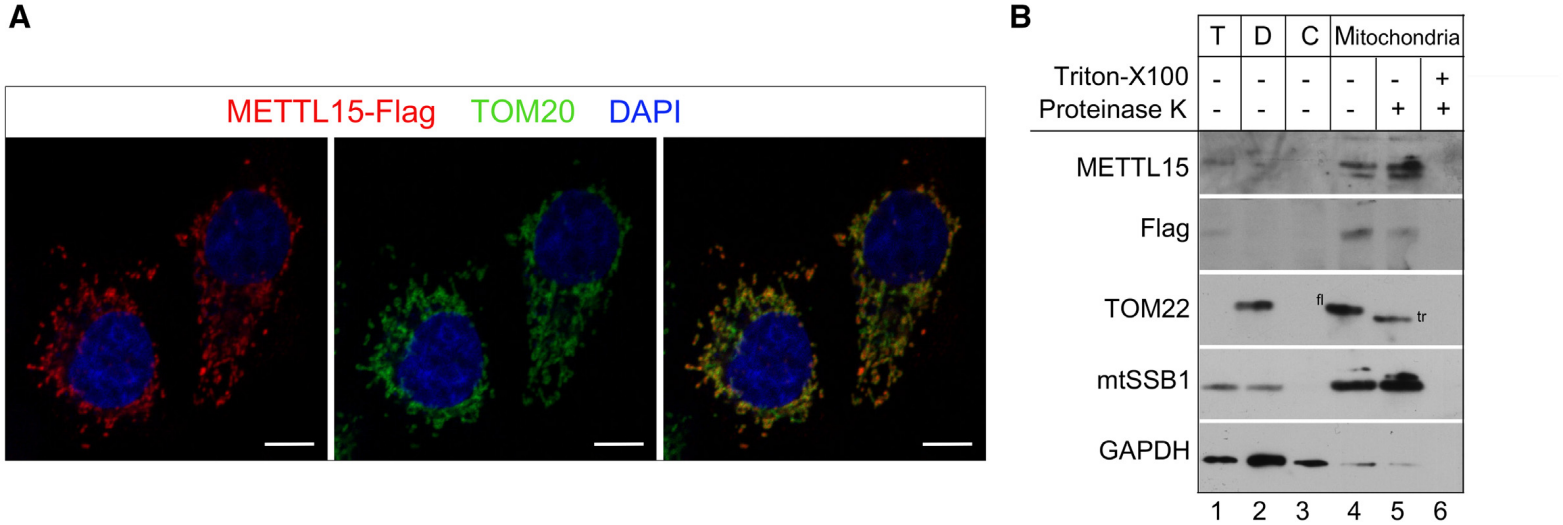
METTL15 localizes in the mitochondrial matrix. (**A**) Immunofluorescence labelling of a Flag-tagged METTL15 construct (red) in HeLa cells. Cells were counterstained for the mitochondrial import receptor subunit TOM20 (green) and DAPI (blue). Scale bar, 10 μm. (**B**) Sub-cellular localisation of METTL15 analysed by western blotting with antibodies against METTL15, Flag, TOM22 (mitochondrial outer membrane), mtSSB1 (mitochondrial matrix) and GAPDH (cytosol). HEK293T cells expressing a Flag-tagged METTL15 construct were fractionated into debris (D, lane 2), cytosol (C, lane3) and mitochondria (M, lane 4–6). ‘T’ indicates the total cell lysate. ‘fl’ indicates full-length TOM22, ‘tr’ stands for truncated TOM22.

To examine if METTL15 is a mitochondrial protein, we transiently expressed a FLAG-tagged version of the protein in HeLa cells and subjected these cells to immunofluorescence detection. In this analysis, METTL15 co-localized with the mitochondrial protein TOM20 in HeLa cells (Figure 1A). Subsequent cellular fractionation experiments of HEK293T cells stably expressing the FLAG-tagged version of METTL15 indicated that METTL15 is enriched within the mitochondrial fraction, along with mtSSB1, a well-characterized mitochondrial matrix protein (Figure 1B). Both mtSSB1 and METTL15 were resistant to proteinase K treatment of the mitochondrial fraction, whereas the outer membrane protein TOM22 was truncated by proteinase K digestion in similar conditions. Although METTL15 has no predicted mitochondrial targeting sequence, our results clearly indicate that METTL15 is an intra-mitochondrial protein in human cells. Our results are consistent with a large-scale proteomic study based on proximity labelling that suggested mitochondrial matrix localization of METTL15 (36), as well as with a recent BioID study suggesting that METTL15 interacts with TRUB2, a mitochondrial matrix pseudouridine synthase (13).

### Mitochondrial translation is impaired in METTL15 deficient cells

**Figure 2.**
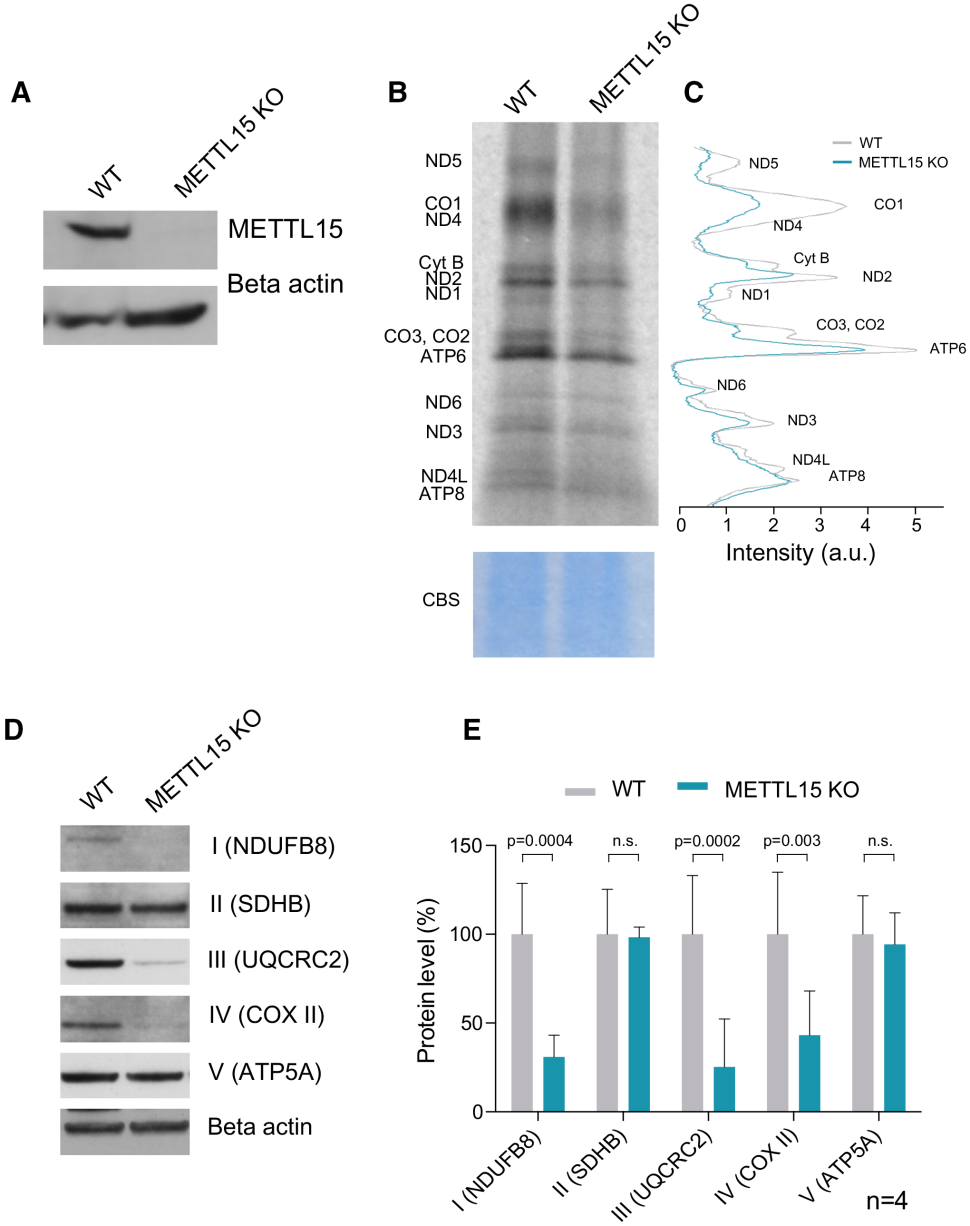
METTL15 is essential for mitochondrial translation. (**A**) Western blot analysis of METTL15 in wild type (WT) and METTL15 KO HAP1 cells. (**B**) Mitochondrial *de novo* protein synthesis was assessed with ^35^S metabolic labelling. Coomassie blue stained (CBS) gel was used as loading control. (**C**) Quantification of the band intensities shown in B using ImageJ. (**D**) Representative example of western blot analysis of NDUFB8, SDHB, UQCRC2, COXII, ATP5A and Beta actin. (**E**) Quantification of 4 western blot experiments for NDUFB8, SDHB, UQCRC2, COXII, ATP5A. Data were statistically analysed by two-tailed Student's *t*-test. Error bars represent standard deviation of the mean.

To study the function of METTL15, the coding gene was knocked-out using CRISPR/Cas9 in HAP1 cells. The METTL15 gene has twelve transcripts, of which six are protein coding (Supplementary Figure S1A). The knockout (KO) cell line carried a 14 bp deletion in exon 3, common to all protein coding transcript isoforms (Supplementary Figure S1A–C). No METTL15 protein was detected in the knockout cells in western blot analysis, consistent with a complete ablation of gene expression (Figure 2A).

To characterize the mitochondrial role of METTL15, we first assessed mitochondrial gene maintenance and expression in the absence of METTL15. We did not detect any appreciable changes in mtDNA copy number in METTL15 KO as compared to wild-type (WT) HAP1 control cells (Supplementary Figure S2A), consistent with mitochondrial biogenesis and maintenance of the mitochondrial genome being unaffected in the absence of METTL15. To test whether the lack of METTL15 affects mt-RNA expression we used qPCR to measure mt-RNA steady-state levels. No statistically significant difference in mitochondrial transcript levels was observed in cells lacking METTL15 (Supplementary Figure S2B). Since RT-qPCR detects mature transcripts as well as precursor mt-RNA, we also assessed mature mt-RNA specifically by northern blot analysis. No significant differences in mature mt-RNA were observed (Supplementary Figure S2C).

**Figure 3.**
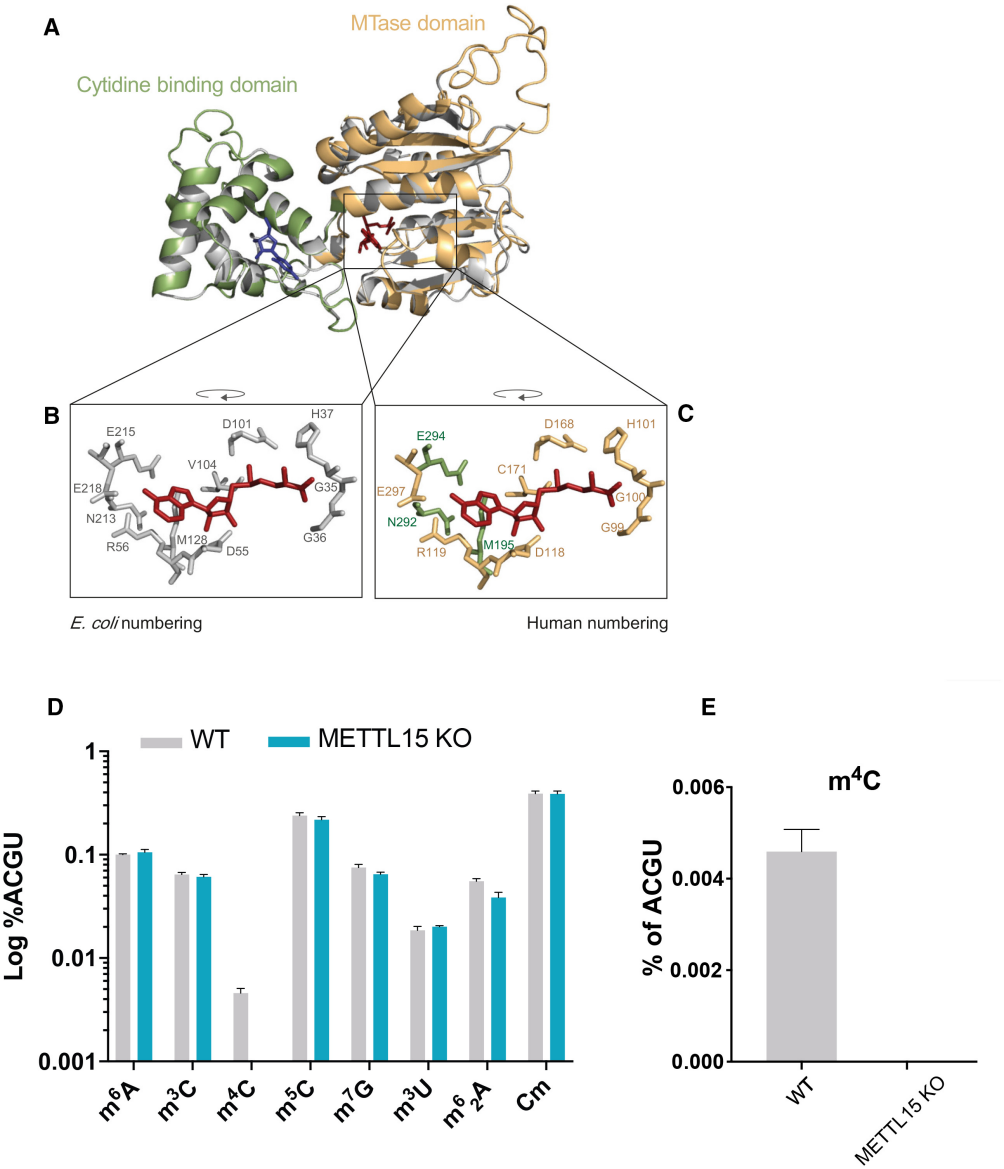
METTL15 is a m^4^C methyltransferase. Structural Analysis of METTL15. (**A**) Homology modelling of *Homo sapiens* METTL15 using *E. coli* RsmH as a template (PDB: 3TKA). The modelled *Homo sapiens* structure is superimposed onto the bacterial structure. Grey, *E. coli* RsmH; green, METTL15 cytidine-binding domain; orange, METTL15 methyltransferase (MTase) domain; red, S-adenosyl methionine; blue, cytidine (**B, C**) Catalytically important residues responsible for AdoMet-binding. Colour coding is as per (A). Homology modelling was performed using Swiss-Model homology modelling server and visualised using PyMol. (D, E) Comparative quantification of various modified nucleosides in mitochondrially-enriched RNA from WT and METTL15 KO HAP1 cells. Nucleosides were quantified by LC-MS, and are expressed as a percentage of the canonical RNA bases. In (**D**), the levels of several modified nucleoside are compared between the cell lines, while in (**E**) the m4C is displayed alone.

We next asked whether the lack of the METTL15 protein affects mitochondrial translation. We measured the rate of mitochondrial protein synthesis by metabolic labelling with radioactively labelled methionine upon inhibition of cytosolic translation. This analysis revealed that the mitochondrial *de novo* protein synthesis rate was generally decreased in the absence of METTL15 (Figure 2B and C). We also performed pulse-chase labelling of mitochondrial *de novo* protein synthesis and observed comparable stability of mtDNA-encoded 7 and 14 h after labelling in WT and METTL15 KO cells. This result suggests that inactivation of METTL15 does not affect translation fidelity and/or membrane insertion of the mtDNA-encoded proteins (Supplementary Figure S3). To confirm this result in a different human model, we silenced METTL15 in HeLa cells using RNA interference (Supplementary Figure S4). Cells treated with siRNA against METTL15 also showed a lower mitochondrial translation rate (Supplementary Figure S4A-B). Furthermore, we asked whether the observed deficiency in mitochondrial translation resulted in reduced levels of respiratory complexes containing mtDNA-encoded subunits. Western blot analysis indicated that the steady-state levels of the protein component of complex I, III and IV were significantly reduced in METTL15 KO cells (Figure 3D and E) and in cells treated with METTL15 siRNA (Supplementary Figure S4C and D). Taken together, our data show that METTL15 is necessary for efficient mitochondrial protein synthesis.

### METTL15 is an m^4^C methyltransferase

The N4-methylcytidine at C1402 (m^4^C1402) modification in the decoding centre of SSU is strongly conserved in bacterial species, including *E. coli* and *Thermotoga maritima* (19–22). Although, m^4^C1402 is regarded as a bacteria-specific modification, recently Leishmania, have also been shown to contain an equivalent SSU rRNA modification (m^4^C2059) (37). Human METTL15 shows sequence homology to bacterial RsmH which introduces m^4^C1402 in the SSU 16S rRNA in *E. coli* (Supplementary Figure S5). C1402 is located in helix 44 (h44) of *E. coli* 16S rRNA and is equivalent to C839 in human 12S mt-rRNA (Supplementary Figure S6); the position that has been previously reported to contain a conserved N4-methylcytidine in mammals (18,38). The *E. coli*, RsmH monomer consists of two structurally related domains: the methyltransferase domain and the cytidine binding-like domain (39). Homology modelling indicated that human METTL15 is also structurally related to RsmH (Figure 3A). The key residues of the active site are conserved between human METTL15 and RsmH, suggesting that the human protein is also an active methyltransferase (Figure 3B-C and Supplementary Figure S5). Given the sequence and structure homology between METLL15 and RsmH, we speculated that METTL15 is responsible for methylation of m^4^C839 in human 12S mt-rRNA. To test this, we examined whether ablation of METTL15 results in changes in m^4^C levels using mass spectrometry (RNA MS). Application of standards showed that in the conditions used RNA MS is able to discriminate between m^3^C, m^4^C and m^5^C (Supplementary Figure S7A). We quantified several modified nucleosides in total RNA preparations from WT HAP1 cells, however, we were not able to detect any signal for m^4^C (Supplementary Figure S7B). Next, we analysed the same set of nucleosides in mitochondria-enriched RNA preparations from WT and METTL15 KO HAP1 cells. Enriching samples with mtRNA allowed for measuring low levels of m^4^C in WT HAP1 cells (Figure 3D, E and Supplementary Figure S7C, grey bars). While most modified RNA nucleosides showed comparable levels in mtRNA-enriched preparations from WT and METTL15 KO cells, m^4^C was present at undetectable levels in the METTL15 KO cells (Figure 3D, E and Supplementary Figure S7C). These results indicate that METTL15 is a homologue of bacterial RsmH and it is a mitochondrial m^4^C methyltransferase. This analysis also shows that mtRNA is the major source of cellular m^4^C, indicating that METTL15 is the main m^4^C methyltransferase in human cells.

### METTL15 methylates 12S mt-rRNA at position C839

**Figure 4.**
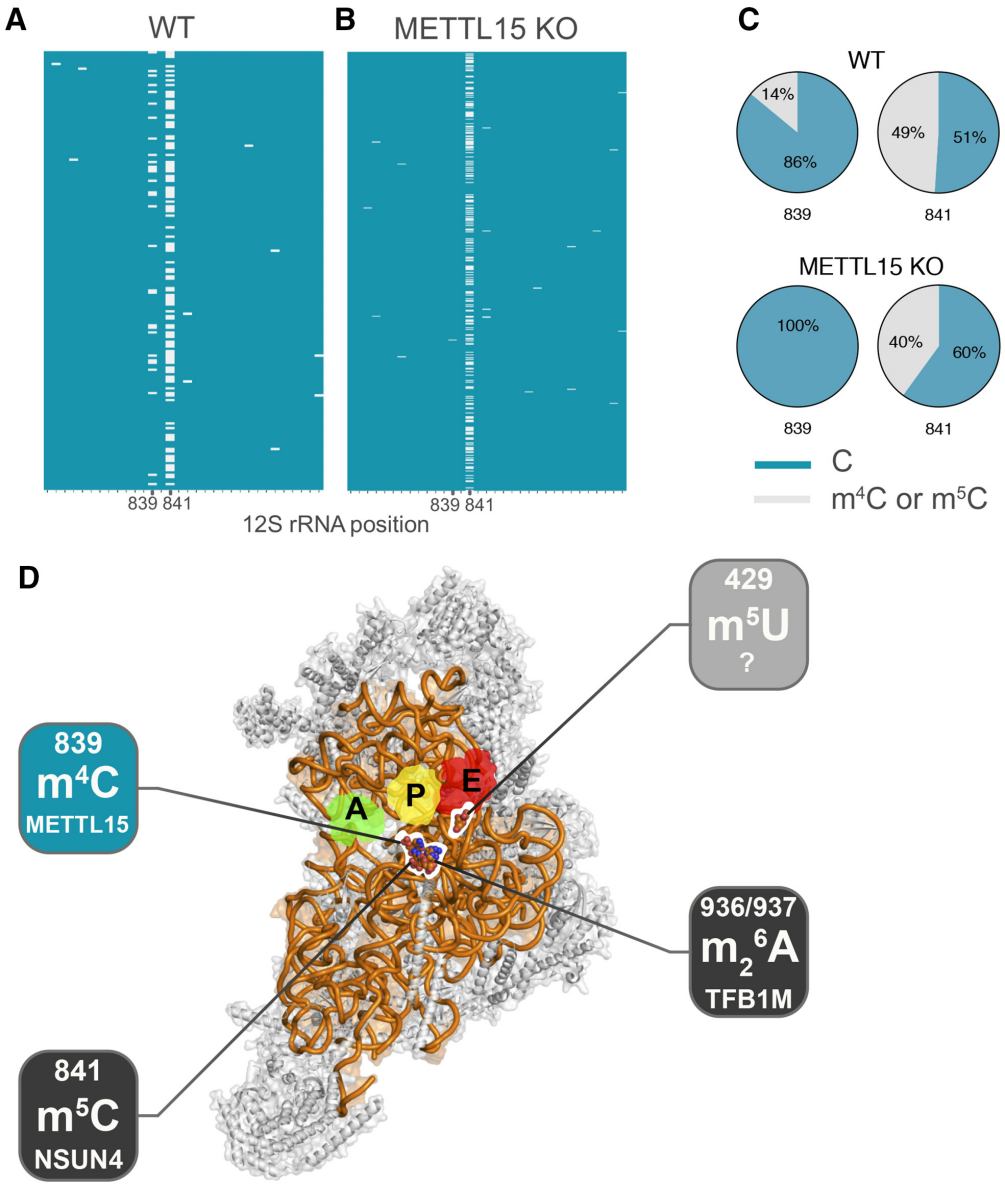
METTL15 methylates position C839 of mitochondrial 12S rRNA. (**A**, **B**) Heatmap of targeted BS RNA-Seq reads for the region of the12S rRNA encompassing position C839 and C841 for wild type HAP1 cells (WT) (A) and METTL15 KO HAP1 cells (B), showing cytosines of individual reads (on y-axis). Methylated cytosines are shown in grey, while unmodified cytosines are shown in blue (x-axis). (**C**) Summary of the targeted BS RNA-Seq results for the C839 and C841 sites in WT and METTL15 KO HAP1 cells. (**D**) View of the human mtSSU from the interface between mitoribosomal subunits. structure of the mtSSU. Putative localisation of tRNAs (green, yellow, red) are presented from structural alignment of a bacterial ribosome loaded with these RNAs (PDB ID: 5JTE). 12S mt-rRNA is presented as an orange ribbon and MRPSs as grey cartoons. 12S mt-rRNA modifications are localised in the three-dimensional, indicating: the position of the known modifications, m^5^C841, m^6^_2_A936 and m^6^_2_A937, for which the enzymes responsible, NSUN4 and TFB1M, respectively, were previously characterised (dark grey), the m^4^C839 modification, for which METTL15 was associated in the present study (teal), and m^5^U429, for which the enzyme remains to be identified (light grey). Adapted from Rebelo-Guiomar *et al.* (45).

Thus far, m^4^C839 in 12S mt-rRNA is the only N4-methycytidine reported in mammalian mitochondria (38,40). In order to investigate if the lack of m^4^C signal in METTL15 KO cells in the RNA MS experiments corresponds to the reduction of m^4^C839 on 12S mt-rRNA we performed targeted RNA bisulfite sequencing (BS RNA-Seq), which combines bisulfite treatment of RNA with high-throughput sequencing. Bisulfite deamination is widely used to study m^5^C (32,41), however, this technique can also be employed to detect m^4^C, although conversion efficacy for m^4^C is lower as compared to m^5^C (42). The BS RNA-Seq analysis confirmed the existence of a modified base at C839 in human 12S mt-rRNA in wild type human HAP1 and HeLa cells, consistent with the presence of m^4^C at this position. This experiment also confirmed the presence of m^5^C841 in human 12S mt-rRNA, known to be methylated by NSUN4 (18) (Figure 4A and Supplementary Figure S4E). Next, we used targeted BS RNA-Seq on RNA samples isolated from METTL15 KO HAP1 cells and compared the methylation of 12S mt-rRNA with WT HAP1 cells. In WT cells we measured approximately 14% of m^4^C839 methylation, whereas in cells lacking a functional METTL15 protein, the m^4^C839 methylation was not detected (Figure 4B, C). In the same analysis, we detected m^5^C841 modification in ∼50% of BS RNA-Seq reads in WT cells, and the signal was reduced to ∼40% in METTL15 KO HAP1 cells (Figure 4B, C). Analogous reduction of the m^5^C841 signal upon inactivation of METTL15, and a consequent lowering of m^4^C839, was observed in the siRNA experiments (Supplementary Figure S4E). This latter observation suggests an interdependence of the m^4^C839 and m^5^C841 modifications. The detected m^4^C839 methylation level of ∼14% in WT HAP1 appears remarkably low compared to other rRNA methylated sites that are usually almost completely modified (11,18,43). This presents an apparent incongruous contrast between the low level m^4^C839 and the strong effect of its absence on mitochondrial translation. However, this potential disparity is likely to be attributed to the low efficiency of m^4^C conversion in BS RNA-Seq. Consequently, the actual C839 methylation levels are likely to be much higher in WT cells, explaining the observed effect in the METTL15 KO cells. Our results are also consistent with the previously published data reporting a degree of m^4^C/m^5^C modification in mouse 12S mt-rRNA (18). Nevertheless, bisulfite conversion clearly shows the absence of N4-methylation of position C839 of human 12S mt-rRNA in cells lacking a functional METTL15 (Figure 4C) and reduced m^4^C levels in cells treated with siRNA against METTL15 (Supplementary Figure S4E), consistent with this enzyme being responsible for introducing m^4^C839 (Figure 4D).

### Catalytic activity of METTL15 is necessary for mitochondrial translation

**Figure 5.**
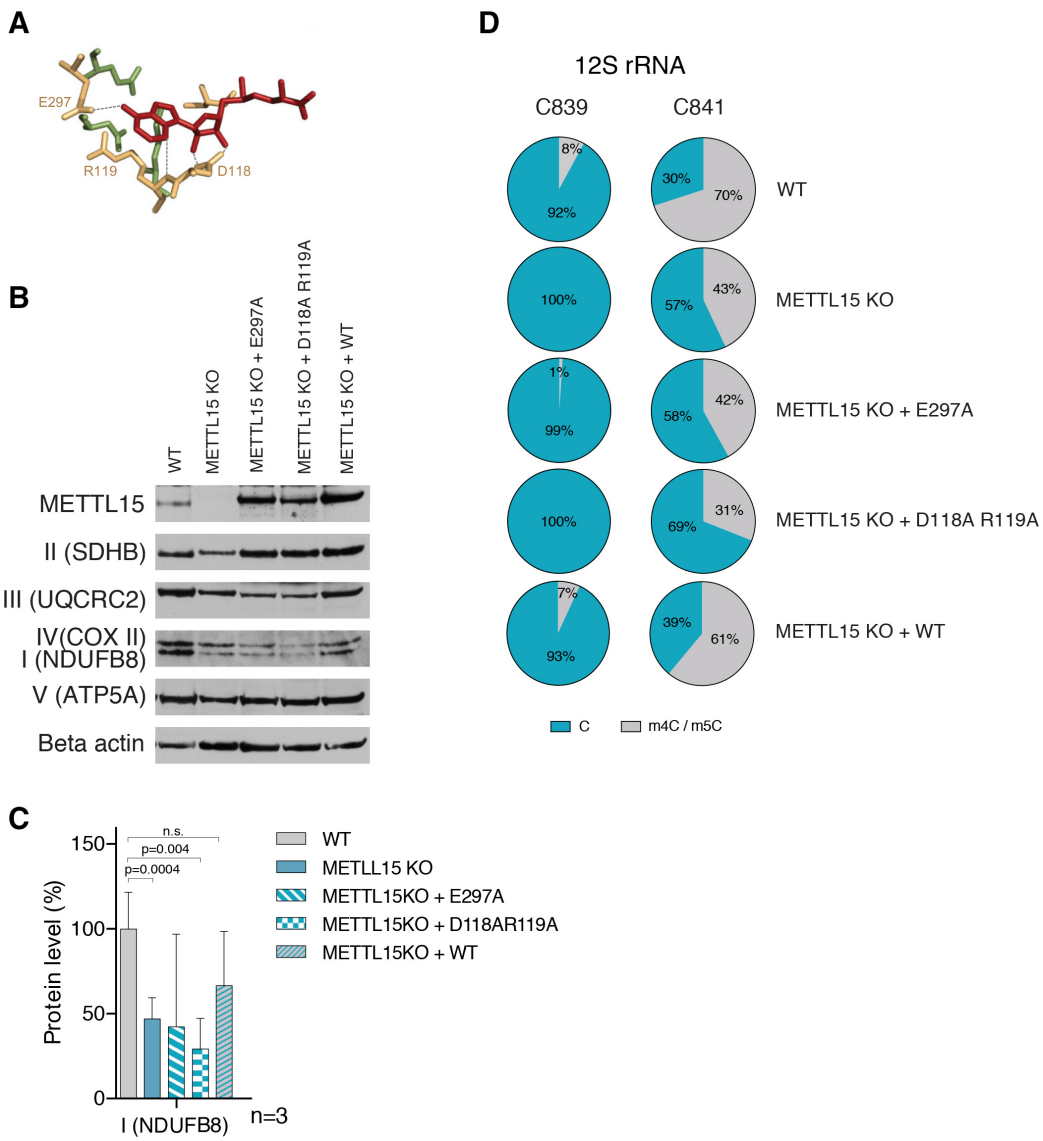
cDNA complementation of METTL15 KO cells.(**A**) Catalytically important residues, D118, R119 and E297, predicted to bind AdoMet (red), that were subjected to site-directed mutagenesis are indicated on the homology model as per Figure 1. The dotted line represents the predicted interactions with AdoMet based on Wei *et al.* (2012). (**B**) Western blot analysis of METTL15, NDUFB8 (complex I), SDHB (complex II), UQCRC2 (complex II), COXII (complex IV), ATP5A (complex V) and Beta actin in wild type HAP1 cells, METTL15 KO cells, METTL15 KO cells transfected with cDNA encoding E297A or D118A + R119A mutants or with WT METTL15 cDNA. CBS stands for Coomassie blue staining. (**C**) Quantification of NDUFB8 analysed by western blot (*n* = 3, data were statistically analysed by two-tailed Student's *t*-test. Error bars represent standard deviation of the mean). (**D**) Pie charts of BS RNA-Seq results showing methylation percentage of 12S mt-rRNA position C839 and C841 in wild type HAP1 cells, METTL15 KO cells, and METTL15 KO cells complemented with E297A, D118A + R119A mutants or with WT METTL15 cDNA.

In order to investigate whether it is the presence of the METTL15 protein, its catalytic activity, or both that are crucial for mitochondrial function we complemented METTL15 KO cells with cDNA encoding wild type or predicted catalytically inactive mutants of METTL15. Two METTL15 mutants were designed: a single mutant carrying E297A and a double mutant harbouring the D118A and R119A amino acid substitutions. The E297 and D118 residues interact directly with AdoMet, whereas R119 has an indirect interaction via well-ordered water molecules (39) (Figure 5A). Following lentiviral transduction and selection, we have confirmed expression of the METTL15 protein and variants thereof. Next, we assessed of steady state levels of OXPHOS complexes by western blotting (Figure 5B-C) and the C839 methylation levels by BS RNA-Seq (Figure 5D). Complementation of METTL15 KO cells with WT METTL15 protein resulted in a clear rescue of both methylation and OXPHOS complex steady state levels. On the other hand, complementation with a mutant METTL15 protein, resulted in only very low (E297A) or no methylation (D118A + R119A) of 12S mt-rRNA position C839 and no detectable correction of OXPHOS complex steady state levels compared to METTL15 KO cells. These experiments additionally confirmed our previous observation that incorporation of m^4^C839 influences modification at C841 (Figure 4B-C, Supplementary Figure S4E), showing that the loss of the C839 modification leads to reduction of the C841 modification (Figure 5D). Taken together these data indicate that the methylation of 12S mt-rRNA at C839 by a catalytically active METTL15 is necessary for mitochondrial translation.

### METTL15 is required for biogenesis of the mitochondrial ribosome

**Figure 6.**
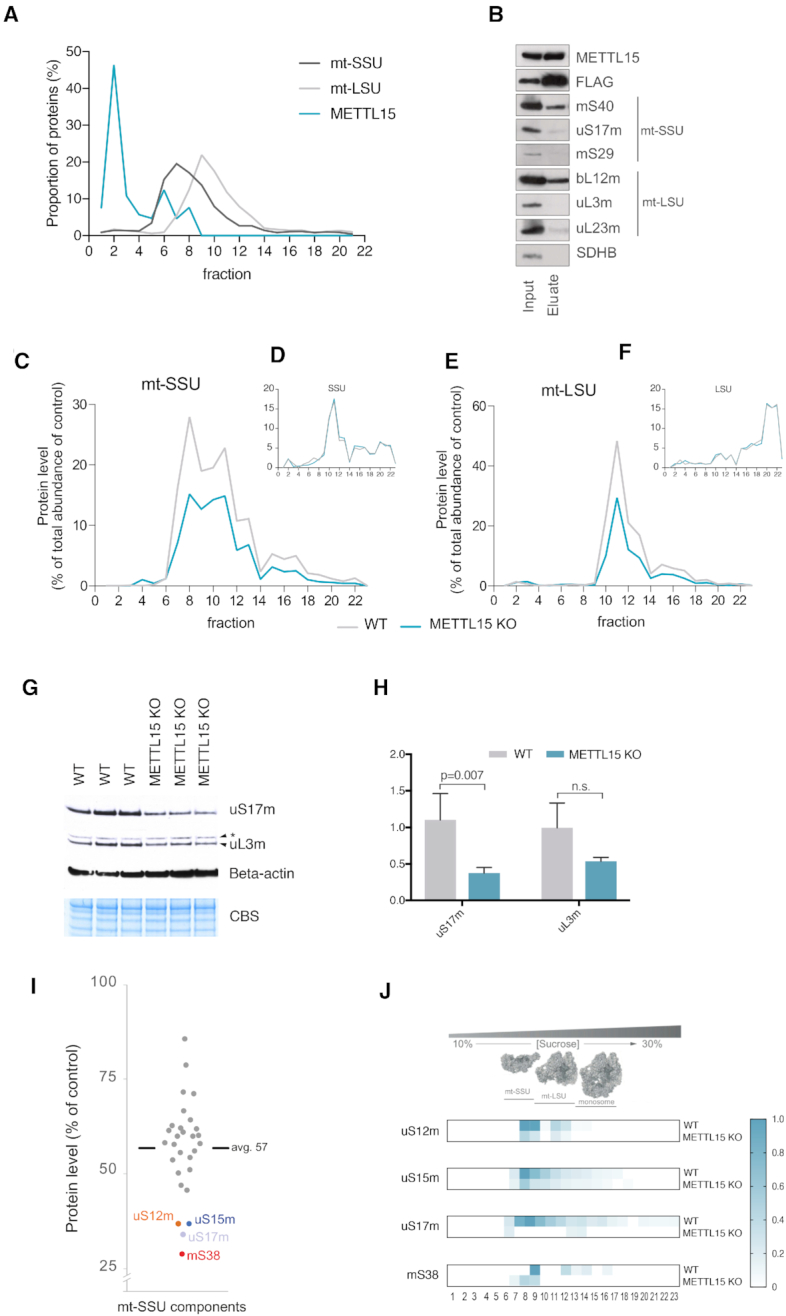
Effect of METTL15 inactivation on the mitochondrial ribosome. (**A**) An average (*n* = 4) proteomic profile of mt-SSU, mt-LSU and METTL15 upon sucrose gradient sedimentation. (**B**) Immunopurification of components of the mitochondrial ribosome using a flag-tagged version of METTL15. HEK293T cells expressing a flag-tagged METTL15 construct were lysed and immunoprecipitation was performed using anti-Flag antibodies. The lysate (Input) and eluates were analysed by western blot with antibodies as indicated. (C–F) A representative quantitative sucrose gradient sedimentation analysed by mass spectrometry. Wild type and METTL15 KO HAP1 cells were grown in heavy or light labelled medium and the different fractions after sucrose gradient sedimentation were analysed by mass spectrometry. Graphical representation of total mt-SSU proteins (**C**) and cytoplasmic SSU proteins (**D**) or mt-LSU proteins (**E**) and cytoplasmic LSU proteins (**F**) detected in each fraction for wild type (grey) or METTL15 KO HAP1 (blue) cells. See also Supplementary Dataset. (**G**) Western blot analysis of three independent wild type and METTL15 KO HAP1 samples for uS17m, uL3m and Beta-actin. CBS stands for Coomassie blue staining. Asterisk represents non-specific bands. (**H**) Quantification of band intensities shown in G (n = 3, data were statistically analysed by two-tailed Student's t-test. Error bars represent standard deviation of the mean). (**I**) Protein steady-state levels of the mt-SSU components in METTL15 KO HAP1 cells, relative to wild type control cells, in the mt-SSU fractions. The most affected proteins are coloured. The results were obtained from duplicate experiments with reciprocal SILAC (Control:heavy/METTL15 KO:light or Control:light/METTL15 KO:heavy) (**J**) Representative example of relative intensity profiles of the mt-SSU proteins detected in METTL15 KO HAP1 that were in the bottom 20% of steady state level values, as compared to the WT control.

We performed a sucrose gradient sedimentation of WT HEK293T cell lysates followed by detection and relative quantification of different components of the mt-SSU and mt-LSU by mass spectrometry to examine the interaction of METTL15 with the mitoribosome. Although the majority of detected METTL15 sedimented in early fractions (fractions 1–4), we did detect partial co-sedimentaton of endogenous METTL15 with mt-SSU (Figure 6A). To corroborate this analysis, we investigated whether METTL15 co-purifies with the mitoribosome. METTL15-FLAG protein was immunopurified from mitochondrial lysates and protein components of both the small and large mitochondrial ribosomal subunit were immunodetected. However, we did not observe any substantial enrichment of mt-SSU or mt-LSU components (Figure 6B). Taken together, these results indicate a weak interaction between METTL15 and the mitoribosome.

We next asked whether the lack of METTL15 affects the integrity of the mitochondrial ribosome. To unravel any potential quantitative changes in the steady-state levels of the assembled mt-SSU and mt-LSU, we combined sucrose gradient sedimentation protein profiling with relative quantification by stable isotope labelling with amino acids in cell culture (SILAC). WT or METTL15 KO HAP1 cells were grown in media containing normal amino acids or amino acids labelled with stable heavy isotopes and subjected to sucrose gradient sedimentation upon mitochondrial enrichment. Mitoribosomal protein components from each fraction were identified by mass spectrometry analysis and quantified in a relative fashion using the observed intensity of a representative peptide (Supplementary Dataset). The protein profiling data showed that the abundance of mitoribosomal proteins was generally reduced by ∼40% in cells lacking METTL15 as compared to WT cells, while cytoplasmic ribosomal proteins are detected at similar levels in both cell lines (Figure 6C–F). These results were validated by western blot analysis of total cell lysates of cells grown in SILAC labelling medium for uS17m and uL3m (Figure 6G and H). This confirmed a decrease in steady-state levels of both mt-SSU and mt-LSU in METTL15 KO cells. Further analysis also revealed a non-statistically significant decrease in mitochondrial transcript steady-levels, but not in mtDNA copy number in this medium (Supplementary Figure S2D–E). Northern blot analysis of mature mt-rRNA showed a non-statistically significant decrease in the 12S/16S ratio in METTL15 KO cells (Supplementary Figure S2F and G). To illustrate the MRP reduction in more detail, we compared the protein amounts of the mt-SSU components in the sucrose gradient fractions containing mt-SSU (fractions 8 and 9) (Figure 6I). The four proteins that were most reduced in METTL15 KO HAP1, as compared to the WT control, were identified as mS38 (AURKAIP1), uS12m, uS15m and uS17m (Figure 6I and J). When the distances of all MRPSs to the C839 residue are calculated, it is notable that the MRPSs most severely affected by METTL15 knock-out are those localized in the closest proximity to the METTL15 target site (Supplementary Figure S8). In the case of mS38, its closest contact with residue C839 is established between the side chain of L131 and the 2′-hydroxyl of the ribose (8.1 Å, between atoms Cδ1 and O2′). The second closest MRP is uS12m, which contacts the phosphate group of C839 via K72 (∼13 Å between atoms Nζ and OP1 or OP2). The reduction of the steady-state levels of these proteins in the mt-SSU fractions, suggests that they cannot incorporate effectively in the assembling mitoribosome. Taken together, our data indicate that the lack of m^4^C839 impairs the assembly of the mt-SSU in human mitochondria and that METTL15 is necessary for mitoribosome biogenesis.

## DISCUSSION

Mitochondrial epitranscriptomics is emerging as an important aspect of studies on the regulation of mitochondrial gene expression, with mt-RNA modifications being important for efficient and accurate protein synthesis in mitochondria in health and disease (44–47). Ten post-transcriptional modifications have been detected in mammalian mitochondrial ribosomal RNAs. Most of these modifications and/or modifying enzymes play a role in mitoribosome biogenesis (9,14,17). The three 2′-*O*-ribose methylations of the large 16S mt-rRNA, Gm1145, Um1369 and Gm1370 are catalyzed by MRM1, MRM2 and MRM3, respectively (8–10). Depletion of MRM2 and MRM3 results in aberrant assembly of the large subunit and diminished mitochondrial protein synthesis. Furthermore, the mitochondrial pseudouridine synthase RPUSD4, suggested to introduce Psi1397 in 16S mt-rRNA, is essential for the stability of the large mitoribosomal subunit (13,14). Modification of the two adjacent dimethylated adenosine residues (m^6^_2_A936 and m^6^_2_A937) by TFB1M in human 12S mt-rRNA has been proposed to act as a checkpoint in the regulation of mitoribosome biogenesis, similar to the bacterial homolog KsgA (48), and the loss of the dimethylation impairs the assembly of mt-SSU and mitochondrial translation (17). Finally, inactivation of NSUN4, which methylates C841 (m^5^C841) of the small 12S mt-rRNA and works in a complex with MTERF4, also affects mitoribosome assembly (18). The present study identifies a novel mt-rRNA modifier: the previously uncharacterized RNA methyltransferase METTL15, which introduces m^4^C839 in the small 12S mt-rRNA.

In *E. coli*, C1402 located in h44 of the small subunit 16S rRNA, corresponds to C839 in human 12S mt-rRNA. The SSU C1402 has a unique methyl modification, N4, 2′-*O*-dimethylcytidine (m^4^Cm) (49), which is conserved in other bacteria (50–52). This N4-methylated cytidine, generated by the METTL15 ortholog RsmH, interacts directly with the P-site codon of the mRNA (53). In the absence of RsmH, non-AUG initiation is increased and stop codon read-through is decreased. The 2′-*O*-methyl modification at C839 is not conserved in mammalian mitochondrial RNA, however, the N4-methyl at this position is strongly conserved. In the mitochondrial ribosome, the m^4^C839 modification is also predicted to be located near the P-site codon of the mRNA, suggesting a role in decoding (Supplementary Figure S8) (1,4). In humans, mitochondrial translation occasionally initiates with AUA (MT-ND1, MT-ND3, MT-ND5) or AUU (MT-ND2) instead of the universal start codon AUG. In accordance with its bacterial function, we suggest that the m^4^C839 methylation plays a role in fine-tuning the shape and function of the P-site and as such increasing decoding fidelity, in particular the correct recognition of the initiation codon, however, more research is needed to support this hypothesis.

**Figure 7.**
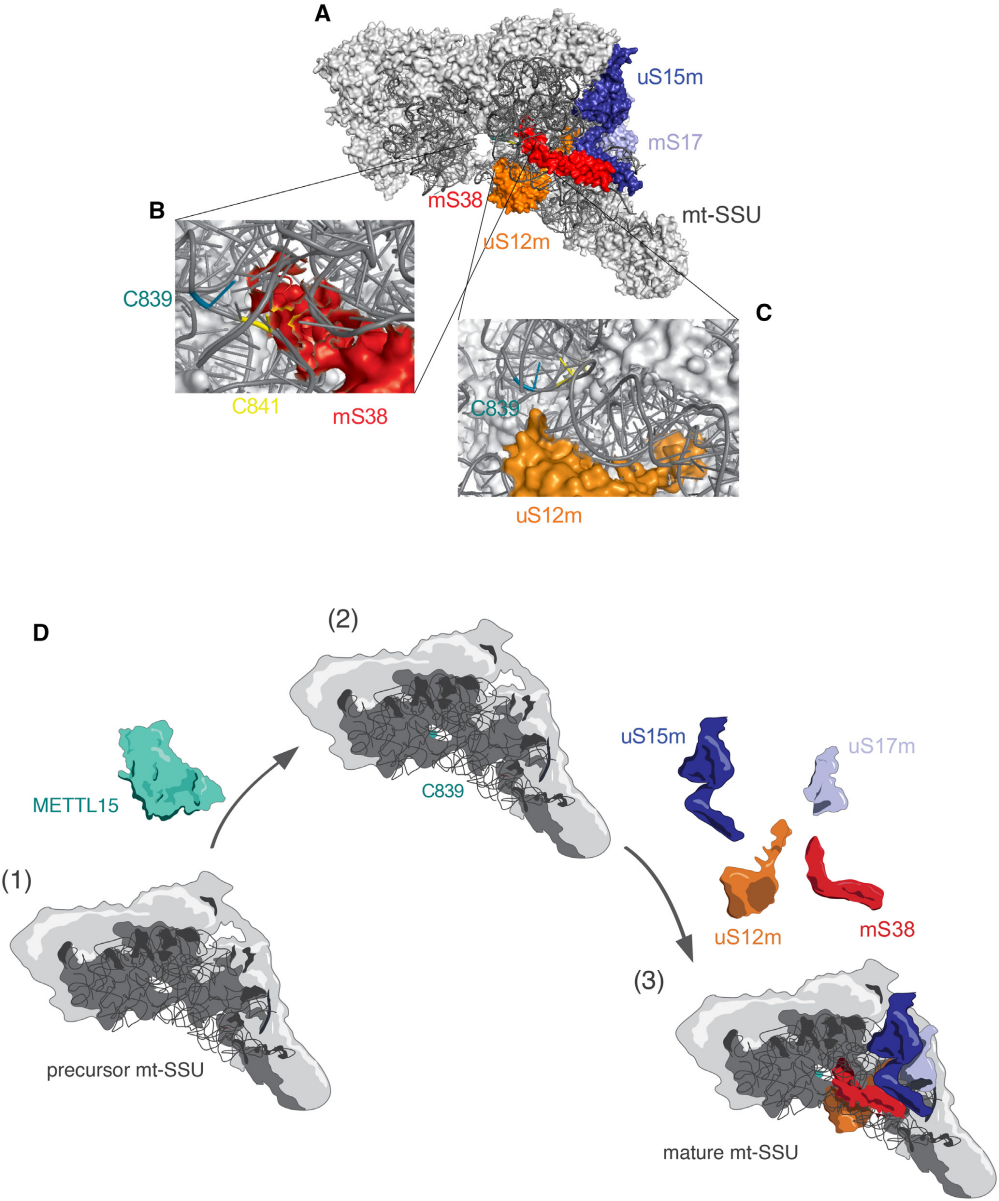
Possible role of methylation of position C839 of mitochondrial 12S rRNA. (**A**) Structure of mt-SSU (PDB: 5AJ4) (4) indicating the proteins that are downregulated in METTL15 KO as compared to WT samples. uS15m in dark blue, uS17m in light blue, mS38 in red and uS12m in orange. (**B**) Detail of (A) showing the localisation of mS38 (red) and mt-rRNA positions C839 and C841. (**C**) Detail of (A) showing the localisation of uS12m (orange) and mt-rRNA positions C839 and C841. (**D**) Model for the role of METTL15 in mt-SSU assembly. (i) Partially assembled mt-SSU. (ii) METTL15 methylates 12S mt-rRNA at C839. Teal sphere denotes the methyl group. (iii) Methylation of C839 introduces a structural change on 12S mt-tRNA and enables the incorporation of late stage ribosomal components into mt-SSU.

It has been shown that bacterial RsmH recognizes the small ribosomal subunit as a substrate, but not the naked rRNA or the tightly-coupled monosome (53). This is consistent with RsmH acting on later stages of biogenesis of the bacterial small ribosomal subunit. The co-migration of the METTL15 pool with mt-SSU (Figure 6A) also suggests that the mitochondrial small ribosomal subunit is a substrate for this enzyme. It has been suggested previously that m^4^C839 and its neighbouring methylated cytidine at position 841 are involved in the stabilization of 12S rRNA folding and thereby facilitating mitoribosomal assembly (18). Our data demonstrate that in the absence of METTL15 the biogenesis of the mt-SSU is generally impaired, with a subset of mt-SSU components (mS38, uS15m, uS17m and uS12m) being affected to a greater degree. However, the partial inhibition of mitochondrial protein synthesis (Figure 2B and C) suggests that fully assembled mt-SSU is also formed in the METTL15 KO cells. The structural data show that mS38 is located in the proximity of 12S mt-rRNA C839 (Figure 7A, B, Supplementary Figure S6) (1,4). The aberrant incorporation of mS38 could be caused by its inability to interact with improperly folded 12S mt-rRNA in the absence of m^4^C839. The uS15m protein is also within the subset of mt-SSU proteins most affected by the absence of METTL15 (Figure 6I and J). The structure of human mt-SSU shows that uS15 is the only mt-SSU protein that interacts with mS38 (1,4), therefore, the assembly of these two proteins could be interdependent. This is consistent with a recent study by Bogenhagen *et al.* (7) on the kinetics of mitoribosome assembly that indicates uS15m as part of a late stage mt-SSU assembly cluster (also including mS25 and mS26) that associates with mS38. Our analysis also shows that assembly of uS12m into the mt-SSU is severely altered in the absence of METTL15 (Figure 6I, J and Figure 7A and C). Since this protein is also located near position C839 (Supplementary Figure S8), this reinforces the notion that methylation of C839 introduces a structural change necessary for uS12m to bind. Furthermore, uS17m, a mt-SSU protein that interacts with both uS15 and uS12m, also shows a more substantial downregulation in the METTL15 KO cells. The kinetic model by Bogenhagen *et al.* suggests that uS12m and uS17m are early binding proteins that incorporate into the mt-SSU as individual proteins. Neither uS12m or uS17m contacts other early-binding proteins, so their addition appears to depend on their substantial interactions with 12S mt-rRNA. Consequently, a potential structural change of the 12S mt-rRNA in the absence of METTL15 could prevent stable protein-RNA interactions of uS12m and uS17m during the progress of mt-SSU biogenesis. Moreover, our experiments showed interdependence of m^4^C839 and the neighbouring m^5^C841, with the loss of the C839 modification resulting in reduction of the C841 modification (Figure 4B, C, Supplementary Figure S4E and Figure 5D). The latter observation could also be related to a potential structural change in h44 of the 12S mt-rRNA, preventing efficient modification of C841 by the NSUN4-MTERF4 complex (18). Taken together, our results provide useful novel information for the study of the mitoribosome assembly process pointing out that METTL15 is required for mt-SSU assembly by regulating local protein-RNA interactions in the vicinity of the C839 site, possibly at the late stages of mitoribosome biogenesis (Figure 7D). Given the decrease in the steady-state levels of mt-LSU in METTL15 KO cells, it would be interesting to explore if METTL15 has other RNA or even protein substrates in addition to 12S mt-rRNA.

A recent study suggested that METTL15 is a part of BioID interactome of a mitochondrial pseudouridine synthase TRUB2. TRUB2 has been shown to interact with several proteins of the mt-LSU as well as with other mitochondrial proteins involved in mt-LSU assembly (13,54). This may indicate that METTL15 could potentially play a role in the mt-LSU biogenesis together with TRUB2. The observed general downregulation of the steady-state levels of mt-LSU in METTL15 KO, in addition to downregulation of mt-SSU, might indeed suggest a role of METTL15 in the production of mitochondrial large subunit. If METTL15 were to be involved in the mt-LSU assembly together with TRUB2, as the interaction detected by BioID suggests, this would have to be a secondary role for METTL15 in mitochondria that needs further investigation, including a detailed characterization of protein-protein interaction using orthogonal methodology.

Many methyltransferases involved in rRNA modification and ribosome assembly have uncoupled (dual) functions i.e. the catalytic activity of an enzyme on rRNA is independent from its role in ribosome biogenesis (e.g. its physical presence in maturing subunits, as a building block of pre-ribosomes). For example, the yeast MRM1 ortholog, Pet56p, has an essential role in the maturation of mt-LSU that is independent from its methyltransferase activity. Also, mammalian NSUN4 is, on the one hand, needed for 12S rRNA methylation and on the other hand, it interacts with MTERF4 to facilitate monosome assembly (18). Our experiments indicate that not only the presence of METTL15, but also its methyltransferase activity is necessary for mitoribosome function (Figure 5A–D). It remains to be investigated whether the function of other mt-rRNA methyltransfereses (e.g. MRM1, MRM2, MRM3, TRMT61B, TFB1M (9–11,28,55)) in mitoribosome assembly is coupled to their catalytic activity.

In summary, we have identified the mitochondrial substrate for the previously uncharacterized N4-methylcytidine RNA methyltransferase METTL15. We demonstrate that this protein is the main human m^4^C methyltransferase and modifies C839 in 12S mt-rRNA. The lack of functional METTL15 protein affects mitoribosomal biogenesis, mitochondrial translation and, consistent with this, mitochondrial OXPHOS performance. Moreover, the function of METTL15 in mitochondrial translation is dependent on its methyltransferase catalytic activity suggesting that the C839 modification itself plays a role in mitoribosome biogenesis and/or stability. The exact molecular role of the m^4^C839 modification in mRNA decoding still remains to be determined. However, since the lack of METTL15 affects assembly of the small subunit of the mitochondrial ribosome and because of its position in the ribosome, we speculate that N4 methylation of position 839, possibly in cooperation with m^5^C841, is involved in stabilization of 12S rRNA folding and thereby facilitating late stages of mitoribosomal assembly.

## Supplementary Material

gkz735_Supplemental_Files
