# Supplementary material for: METTL15 introduces N4-methylcytidine into human mitochondrial 12S rRNA and is required for mitoribosome biogenesis
Source: Nucleic Acids Res. 2019 Sep 6;47(19):10267–81. doi: 10.1093/nar/gkz735 (PMC6821322; doi:10.1093/nar/gkz735)
Supplement: gkz735_Supplemental_Files [file gkz735_supplemental_files.zip › METTL15 Suppl Info 240719 final.pdf]

Supplementary Information

METTL15 methylates mitochondrial 12S rRNA and is required for mitoribosome biogenesis

Lindsey Van Haute *et al*.

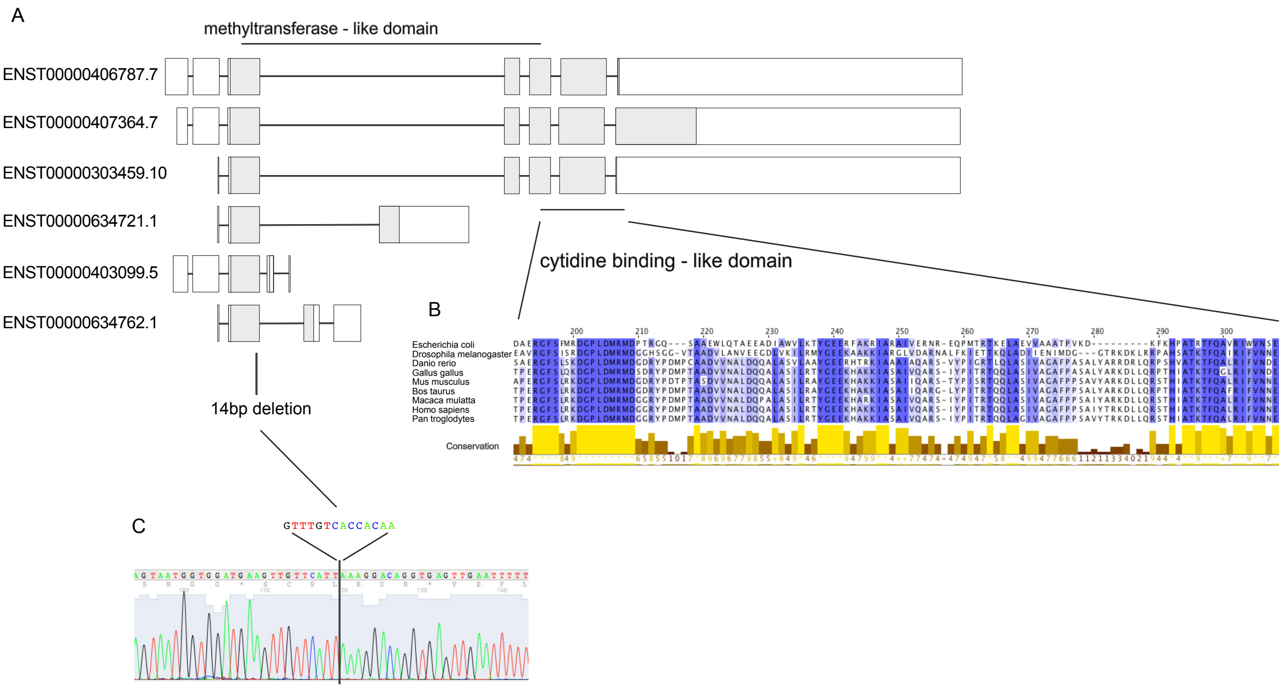

Supplementary Figure 1. (A) METTL15 has six protein coding transcripts. The actual coding part in indicated in grey. (B) Alignment detail for the cytidine binding-like domain. (C) Sanger sequencing results of the mutation analysis of the METTL15 KO HAP1 cell line used in this study.

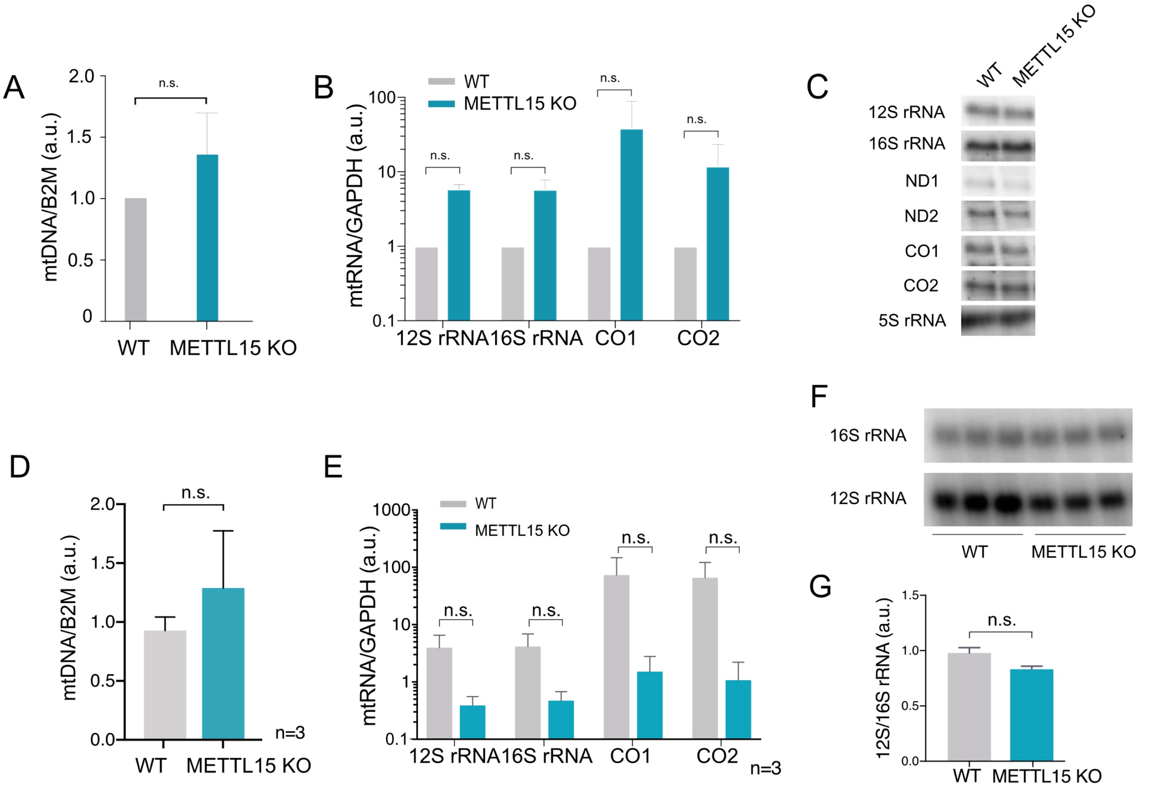

Supplementary Figure 2. (A) mtDNA copy number determination by qPCR of mtDNA fragments relative to the nuclear B2M gene in HAP1 cells (WT and METTL15 KO) grown in regular IMDM medium supplemented with a standard 10% FBS. n=3, error bars indicate the s.d. of the mean. Statistical analysis was carried out using a two-tailed Student's t-test. (B) qPCR determination of transcripts levels in HAP1 cells (WT and METTL15 KO) grown in regular IMDM medium supplemented with a standard 10% FBS. Steady-state levels of mt-rRNA (12S rRNA and 16S rRNA) and selected mt-mRNA (CO1 and CO2) were normalized to GAPDH. n=3. error bars indicate the s.d. of the mean. Statistical analysis was carried out using a two-tailed Student's t-test, (C) Northern blot analysis of 12S rRNA, 16S rRNA, MT-ND1, MT-ND2, MT-CO1, MT-CO2, using 5S rRNA as loading control in HAP1 cells (WT and METTL15 KO) grown in regular IMDM medium supplemented with a standard 10% FBS. (D) mtDNA copy number determination by qPCR of mitochondrial DNA fragments relative to the nuclear B2M gene of cells grown in IMDM medium for SILAC supplemented with Arg, Lys and Pro and 10% dialysed FCS. qPCR was performed in triplicate and error bars indicated the s.d. of the mean. Statistical analysis was carried out using a two-tailed student's t-test. (E) qPCR determination of cells grown in IMDM medium for SILAC supplemented with Arg, Lys and Pro and 10% dialysed FCS of mt-rRNA (12S rRNA and 16S rRNA) and mt-mRNA (CO1 and CO2) compared to GAPDH. Student's t-test. qPCR was performed in triplicate, error bars indicate the s.d. of the mean. (F) Northern blot analysis of 12S rRNA and 16S rRNA in WT and METTL15 KO HAP1 cells (in triplicate). (G) Quantification of band intensities shown in F.

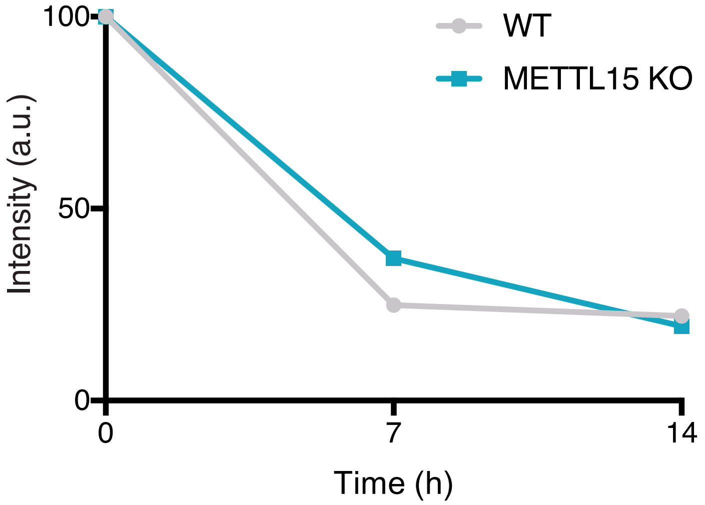

Supplementary Figure 3. Pulse-chase 35S-Met labelling experiment for WT and METTL15 KO cells. Mitochondrial *de novo* protein synthesis was assessed with 35S-Met metabolic labelling and analysed immediately after labelling (0h) or seven and fourteen hours later (n=2). The graph represents the intensity of the mitochondrial protein label incorporation at different time points relative to the intensity at 0h for both WT and METTL15 KO cells.

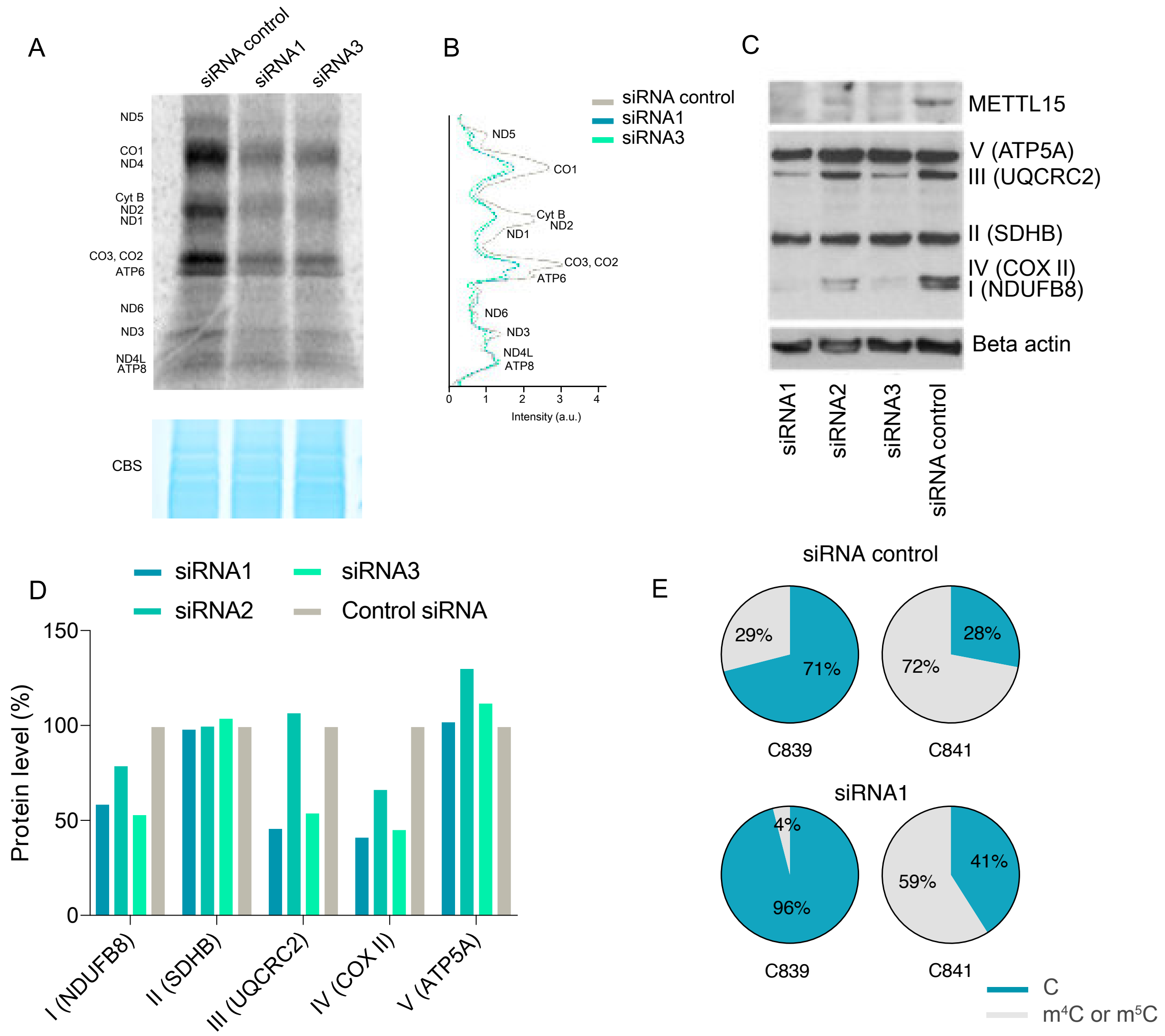

Supplementary Figure 4. (A) Mitochondrial *de novo* protein synthesis was assessed with ^{35}S metabolic labelling in HeLa cells treated with siRNA against METTL15 or control siRNA. Coomassie blue stained (CBS) gel was used as loading control. (B) Quantification of the band intensities shown in A using Image J. (C) Representative example of western blot analysis of METTL15, ATP5A, UQCRC2, SDHB, NDUFB8 and beta actin on HeLa cells treated with siRNA against METTL15 or control siRNA. (D) Quantification of C. (E) Methylation percentage of 12S rRNA position C839 and C841 for Hela cells treated with siRNA against METTL15 or control siRNA.

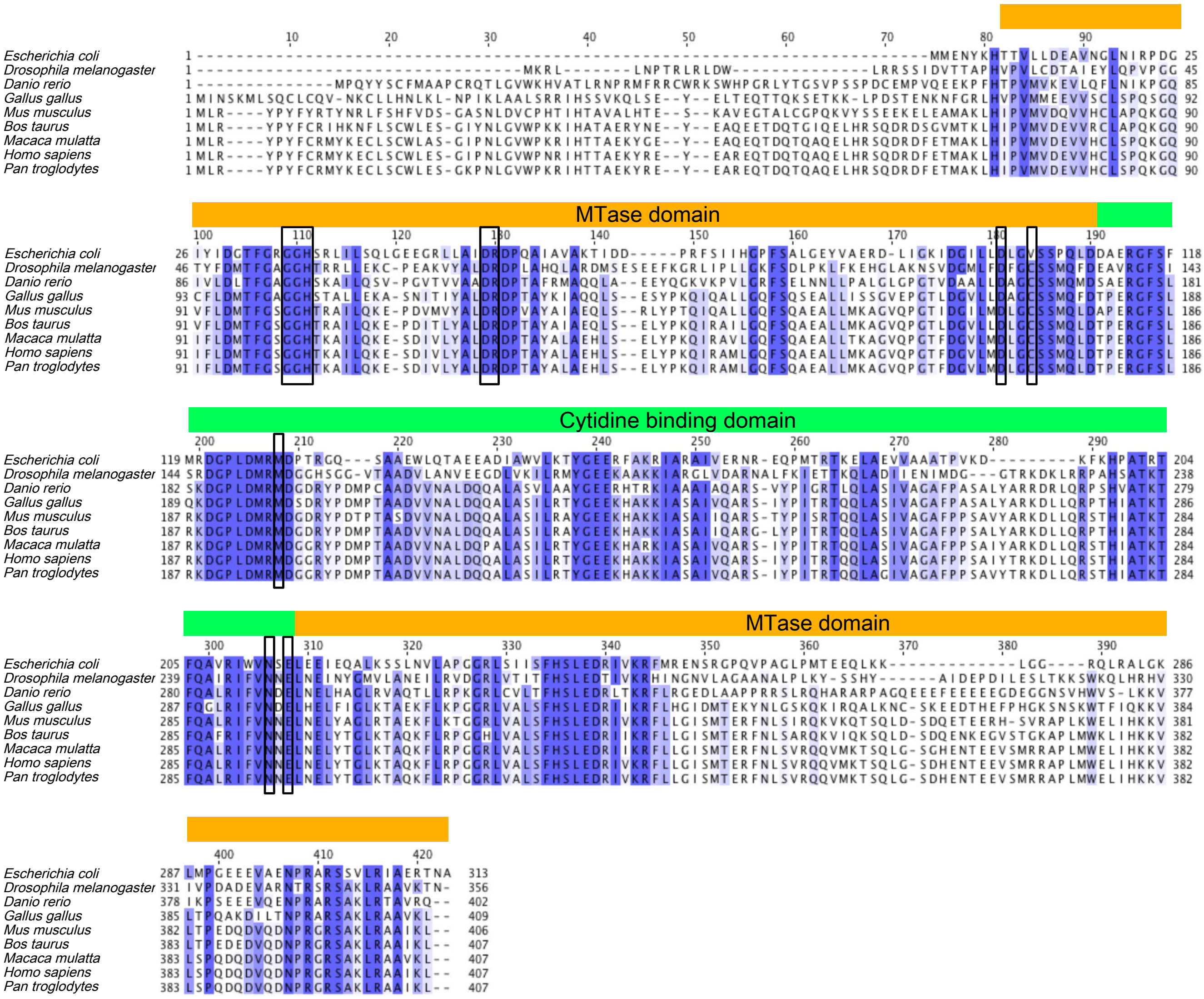

Supplementary Figure 5. Sequence alignment for human METTL15 and a set of homologs including *Escherichia coli*, *Drosophila melanogaster*, *Danio rerio*, *Gallus gallus*, *Mus musculus*, *Bos taurus*, *Macaca mulatta* and *Pan troglodytes*, performed using Jalview. The conserved amino acids are highlighted. The active sites indicated in **Fig. 3B** in the main text are highlighted by a black box.

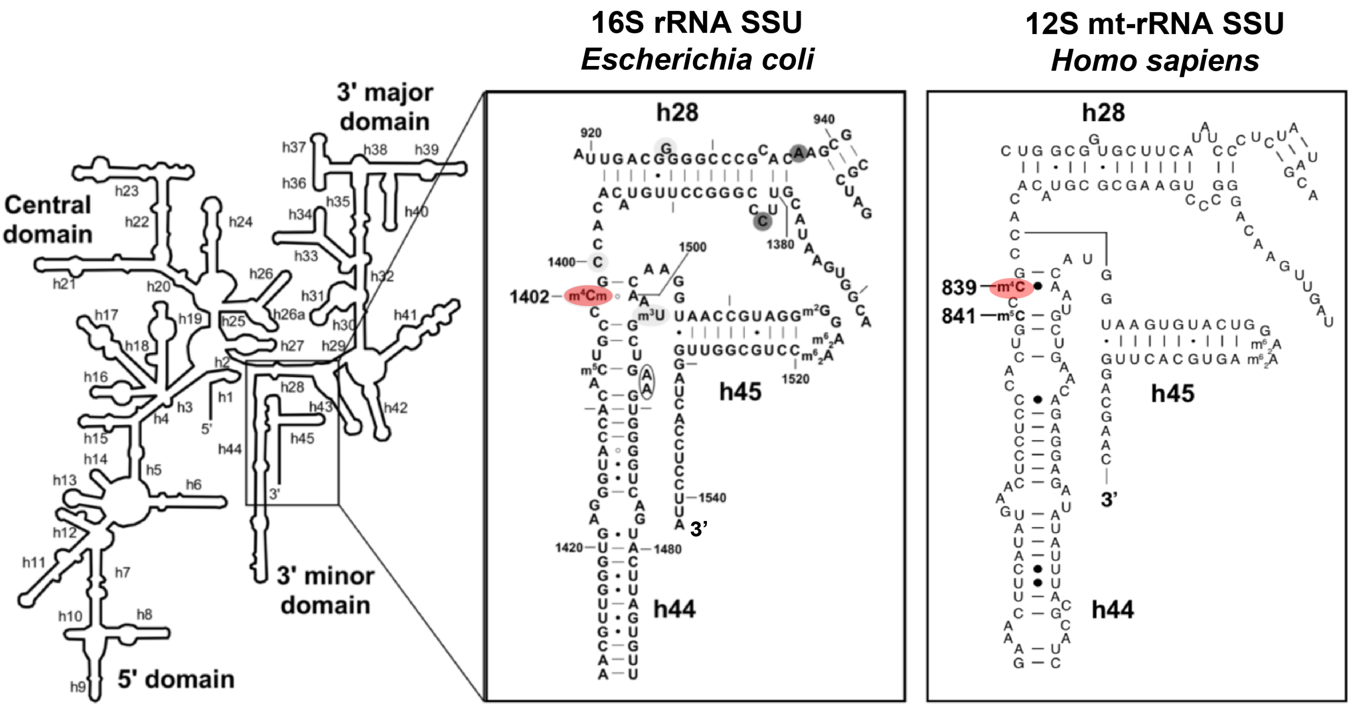

Supplementary Figure 6. Localisation of m^4C sites (red) in the secondary structures of SSU rRNA from *E. coli* (16S) and human mitochondria (12S). Secondary structures were retrieved from Kimura *et al*. (2010, Nucleic Acids Res, 38, 1341–1352) and Amunts *et al*. (2015, Science, 348, 95–98). Figure adapted from Kimura *et al*.

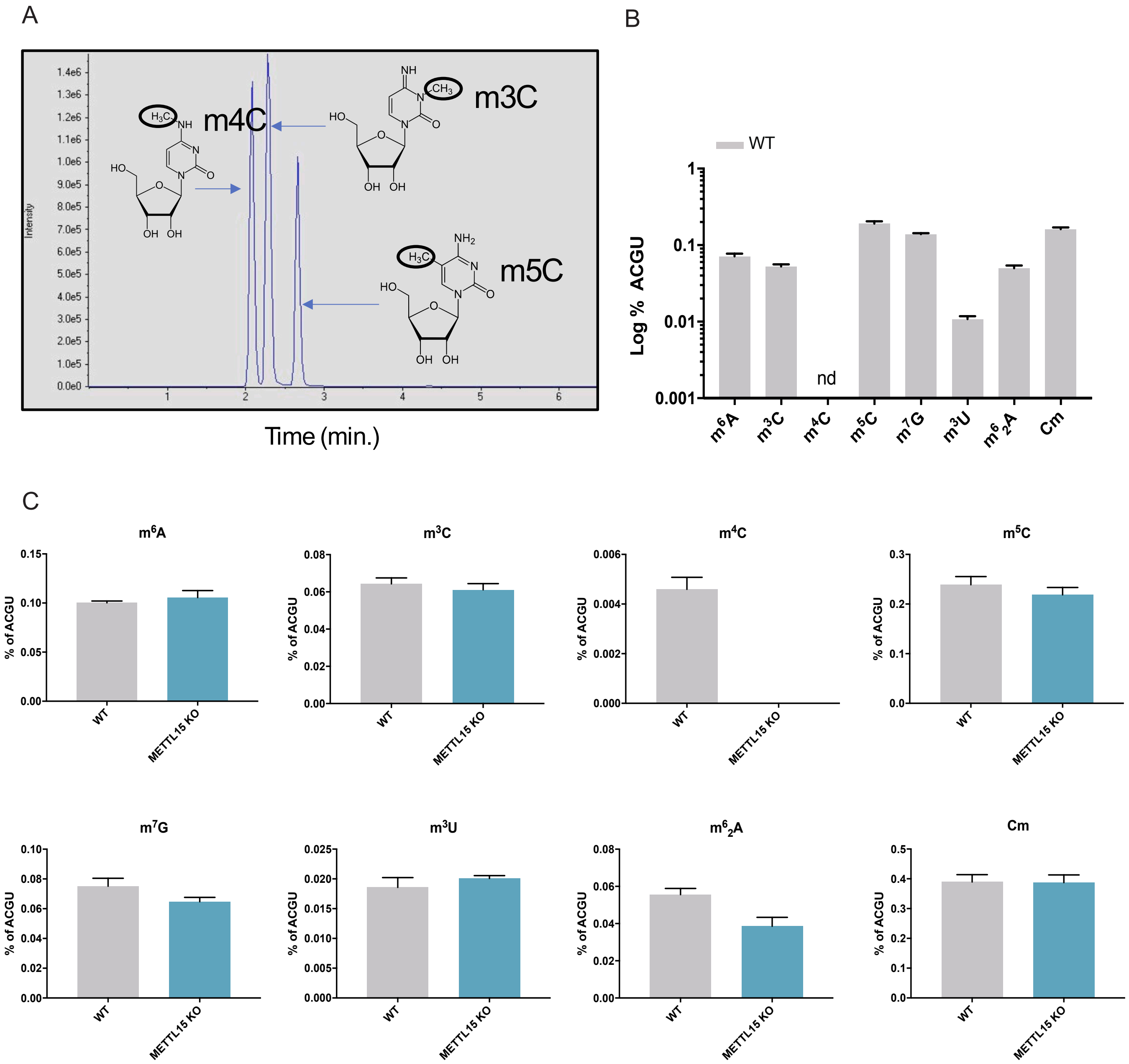

Supplementary Figure 7. Resolution of purified standards of m^{3}C, m^{4}C and m^{5}C by LC-MS. (B) Comparative quantification of various modified nucleosides in total RNA preparations from WT HAP1 cells. Nucleosides were quantified by LC-MS, and are expressed as a percentage of the canonical RNA bases. (C) Quantification of eight modified nucleosides in mitochondrially-enriched RNA by LC-MS. This is the same data as in figure 3, expressed individually with linear axes.

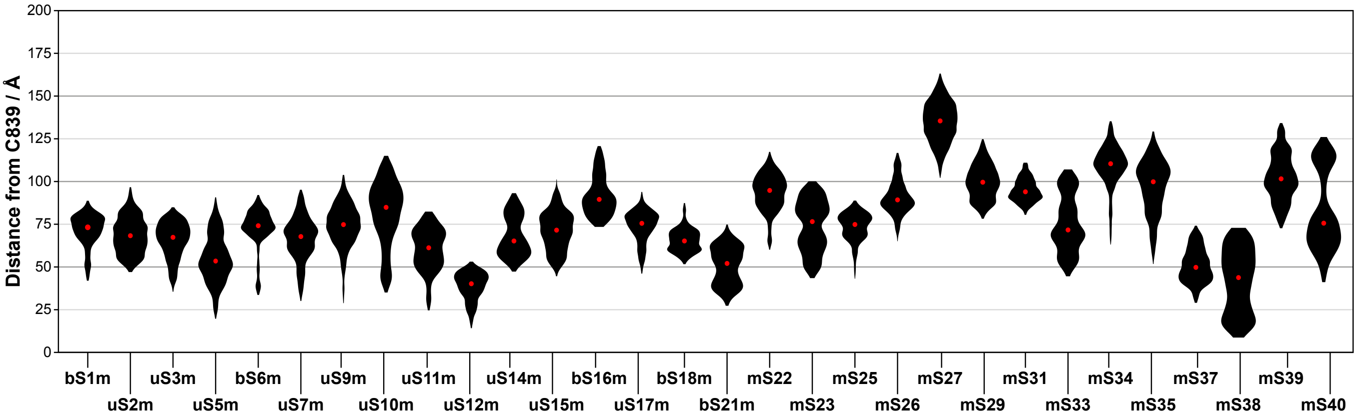

Supplementary Figure 8. Distance of all proteins of mt-SSU to the METTL15 target site as a violin plot representation of atomic distances from C839 in 12S rRNA. The red dot indicates the median distance for each MRPS.

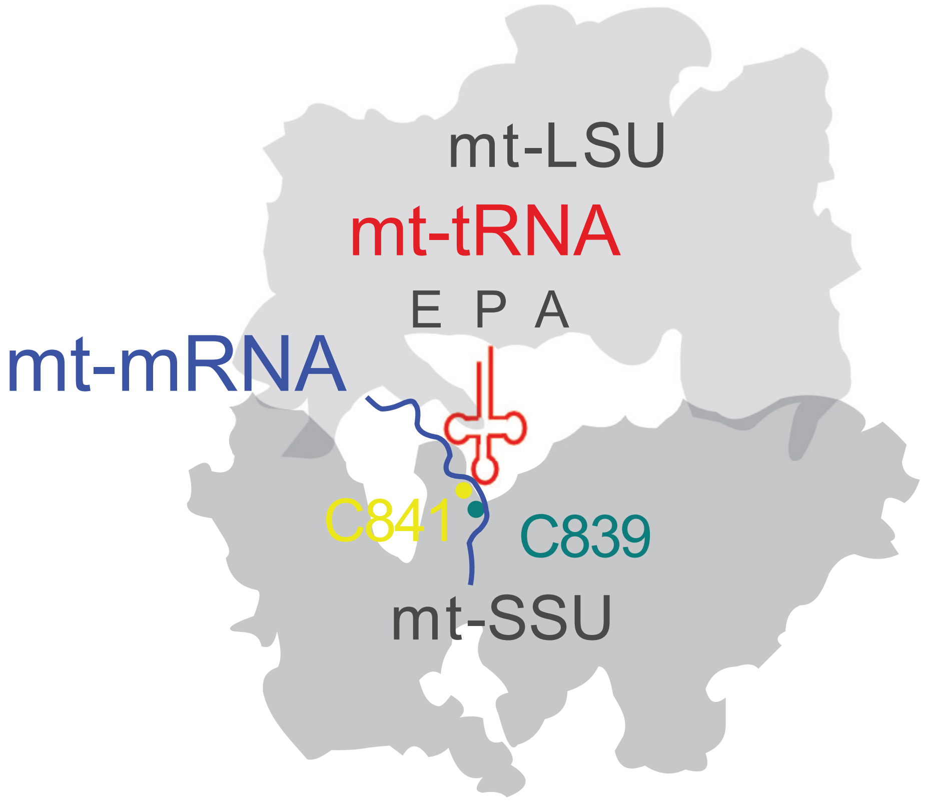

Supplementary Figure 9 Schematic overview of the mitochondrial ribosome. A mt-tRNA at the P site is indicated in red and mt-mRNA is in depicted in blue. The position of C839 and C841 are indicated (mt-rRNA is not indicated for clarity).

Supplementary Table S1: Oligonucleotides used in this study

Please see Excel spreadsheet.

Supplementary Dataset Sucrose gradient sedimentation analysed by mass spectrometry. Wild type (wt) and METTL15 KO (ko) HAP1 cells were grown in heavy (H) or light (L) labelled medium and the different fractions after sucrose gradient sedimentation were analysed by mass spectrometry. The first two spreadsheets show normalised data for two independent datasets. The next spreadsheets show all MRPs for the WT sample and the METTL15 KO sample separately for both datasets.
